# One-Week Exposure to a Free-Choice High-Fat High-Sugar Diet Does Not Interfere With the Lipopolysaccharide-Induced Acute Phase Response in the Hypothalamus of Male Rats

**DOI:** 10.3389/fendo.2018.00186

**Published:** 2018-04-30

**Authors:** Evita Belegri, Leslie Eggels, Susanne E. la Fleur, Anita Boelen

**Affiliations:** ^1^Department of Endocrinology and Metabolism, Academic Medical Centre, Amsterdam, Netherlands; ^2^Laboratory of Endocrinology, Department of Clinical Chemistry, Academic Medical Centre, Amsterdam, Netherlands; ^3^Metabolism and Reward Group, Netherlands Institute for Neuroscience, Royal Netherlands Academy of Arts and Sciences (KNAW), Amsterdam, Netherlands

**Keywords:** lipopolysaccharide, diet, obesity, cytokines, hypothalamus, TLR4, endoplasmic reticulum stress

## Abstract

Obesity has been associated with increased susceptibility to infection in humans and rodents. Obesity is also associated with low-grade hypothalamic inflammation that depends not only on body weight but also on diet. In the present study, we investigated if the bacterial endotoxin [lipopolysaccharide (LPS)]-induced acute phase response is aggravated in rats on a 1-week free-choice high-fat high-sugar (fcHFHS) diet and explained by diet-induced hypothalamic inflammation. Male Wistar rats were on an fcHFHS diet or chow for 1 week and afterwards intraperitoneally injected with LPS or saline. Hypothalamic inflammatory intermediates and plasma cytokines were measured after LPS. Both LPS and the fcHFHS diet altered hypothalamic *Nfkbia* mRNA and nuclear factor of kappa light polypeptide gene enhancer in B cells inhibitor alpha (NFKBIA) protein levels, whereas *Il1*β, *Il6*, and *Tnf*α mRNA expression was solely induced upon LPS. We observed an interaction in hypothalamic *Nfkbia* and suppressor of cytokine signaling (SOCS) 3 mRNA upon LPS; both were higher in rats on a fcHFHS diet compared with chow animals. Despite this, plasma cytokine levels between fcHFHS diet-fed and chow-fed rats were similar after LPS administration. Consuming a fcHFHS diet but not LPS injections increased hypothalamic *Atf4* (a cellular stress marker) mRNA expression, whereas *Tlr4* mRNA was decreased only upon LPS. Our study does not support a role for diet-induced mild hypothalamic inflammation in the increased susceptibility to infection despite altered *Nfkbia* and Socs3 mRNA expression after the diet. Additional factors, related to increased fat mass, might be involved.

## Introduction

Obesity is shown to be associated with increased susceptibility to post-operative infections in humans (1–4) and experimental induced infection in rodents (5–7). Lipopolysaccharide (LPS) is a bacterial endotoxin and widely used to investigate the acute phase response upon infection in rodents (6–10). Pohl et al. (6) found that rats, exposed to a high-energy diet that rendered them obese, showed changes in the LPS-induced acute phase response characterized by a prolonged fever response, increased expression of hypothalamic IL1, suppressor of cytokine signaling (SOCS) 3 and IL6 and increased circulating cytokine levels. The hypothalamus is a brain region that plays an important role in the acute phase response *via* inducing behavioral changes (fever, weight loss, and reduced locomotor activity) (8).

We recently showed that short-term exposure to 1-week free-choice high-fat high-sugar (fcHFHS) diet results in mild hypothalamic inflammation characterized by the activation of nuclear factor kappa-light-chain-enhancer of activated B cells (NF-κB) signaling (11). NF-κB is also one of the mediators involved in the acute phase response upon infection *via* degradation of the nuclear factor of kappa light polypeptide gene enhancer in B cells inhibitor alpha (NFKBIA), allowing activation of NF-κB by phosphorylation (p-NF-κB). These events lead to increased mRNA and protein expression of cytokines and the negative feedback regulators of inflammatory signaling SOCS3 (12, 13).

Diet-induced obesity results in a myriad of changes throughout the body, such as inflammation in adipocytes (14, 15), and in the brain (16–18). Both these effects could be involved in the exacerbated immune response during obesity upon infection. It is, however, difficult to distinguish between metabolic changes due to obesity or direct effects of fat and sugar from the diet in studies in obese rats (6, 7), as the animals are exposed to a high-palatable diet for a prolonged period resulting in 15% difference in body weight gain and excessive fat accumulation (6). Interestingly, a recent study showed a hypothalamic inflammatory response already after 3 days of high-fat diet feeding (19) before animals became obese. In addition, we observed with 1-week exposure to a fcHFHS diet, a similar hypothalamic inflammatory response which was linked to nutrient intake (20). Taken together, these studies point to a role for the nutrients in the low-grade hypothalamic inflammation. It could well be that the presence of mild hypothalamic inflammation, induced by the fcHFHS diet, affects the LPS-induced acute phase response.

The aim of the present study was, therefore, to study the LPS-induced acute phase response in male rats after 1-week exposure to a fcHFHS diet compared with rats exposed to chow. The inflammatory response in the hypothalamus was measured 2 h after LPS intraperitoneal (IP) administration and determined by the mRNA expression of inflammatory cytokines (*Il1*β, *Il6, Tnf*α, and *Il10*) and *Socs3* as well as *Nfkbia*, endoplasmic reticulum (ER) stress markers and NFKBIA protein expression. We also measured plasma cytokine and leptin levels at specific time points after an IP injection of LPS.

## Materials and Methods

### Animals

Adult male Wistar rats (250–280 g; Charles River, Germany) were individually housed and maintained at a temperature 19 ± 1°C on a 12 h light/12 h dark cycle, lights on at 7:00 a.m. During the experiments control animals had *ad libitum* access to water and standard laboratory chow (Teklad global diet 2,918, 18.6% protein, 44.2% carbohydrate, and 6.2% fat, 3.1 kcal/g, Envigo, UK). Rats on the fcHFHS diet had simultaneous *ad libitum* access to the standard low-caloric diet, a bottle of tap water, a bottle with 30% sugar water (1.0 M sucrose mixed from commercial grade sugar and water; 1.2 kcal/g) and a dish with pure saturated fat (beef tallow; Ossewit/Blanc de Boeuf, 9 kcal/g, Vandermoortele, Belgium). All the studies were approved by and performed according to the regulations of the Committee for Animal Experimentation of the Academic Medical Center of the University of Amsterdam, Netherlands.

### Animal Experiments

To test the effect of an inflammatory stimulus on the diet-induced changes in the hypothalamus we performed two experiments: (1) we studied the interaction between the diet and the LPS-induced acute phase response at the level of the hypothalamus and (2) we studied the changes in the systemic acute phase response upon LPS in animals on chow or fcHFHS diet.

In experiment 1, 32 male rats were divided into two groups, fcHFHS and control (chow *ad libitum*). After 1 week on the fcHFHS diet or chow diet, each group was divided randomly into two subgroups, LPS and saline. Between 9:00 and 10:00 a.m. animals in the LPS group were injected IP with 100 µg/kg LPS (*Escherichia Coli*, O127:B8, Sigma Aldrich, St. Louis, MO, USA) and those in the saline group with sterile saline. Two hours after LPS or saline injection rats were decapitated under anesthesia (30% CO_2_/70% O_2_). Brains were immediately removed frozen on dry ice and stored in −80°C.

In experiment 2, 20 animals underwent surgery for catheterization of the jugular vein as previously described by Steffens (21). The rats were anesthetized with an IP injection of 80 mg/kg Ketamine (Eurovet Animal Health), 8 mg/kg Rompun^®^ (Bayer Health Care), and 0.1 mg/kg Atropine (TEVA-Pharmachemie), after 1 week of recovery rats were divided into two groups, fcHFHS diet and control (chow *ad libitum*). After 1 week on chow or fcHFHS diet, food was removed and all rats were injected IP with 100 µg/kg LPS between 9:00 and 9:30 a.m. Blood samples (300 µl) were drawn *via* the jugular catheter at time points: 0 min (just before LPS injection), 30 min, 1, 2, 4, and 8 h. Plasma was isolated *via* centrifugation at 3,000 rpm for 15 min at 4°C and stored at −20−C. Eight hours after LPS injection animals were killed using an overdose of pentobarbital sodium and decapitated.

### Plasma Cytokine Measurements

Plasma IL1β, IL10, IP10, TNF-α and leptin concentrations were determined using a rat cytokine ProcartaPlex assay (Bender MedSystems GmbH, Vienna, Austria) and the Bioplex 200 system (BioRad) with an intra-assay variation coefficient of 1.8%, a detection limit of 1 pg/ml, and a calibration range of 1–100,000 pg/ml for all assays. Samples were measured in duplicates and individual values were calculated according to a calibration curve, which was generated using recombinant cytokines diluted in kit matrix for plasma samples.

### Isolation of the Hypothalamus

Coronal brain slices of 250 µm were obtained from 0.96 to 4.36 mm Bregma (Rat atlas, George Paxinos, and Charles Watson) (22) and directly put in RNA later solution (Ambion, Thermofisher Scientific) in a petri dish. The hypothalamic part in each section was dissected and split in two parts at the point of the third ventricle. One part was used for protein detection and the other for mRNA measurements. Left and right hypothalamic areas were randomly assigned for mRNA or protein measurements.

### Western Blotting

Half of the hypothalamus was homogenized in RIPA buffer (50 mM Tris HCL, pH = 7.6, 150 mM NaCl, 1% Triton ×100, 0.5% Sodium Desoxycholate, and 0.1% SDS, 2 mM EDTA), enriched with protease (Complet EDTA-free protease inhibitor cocktail tablets, Roche) and phosphatase (PhosSTOP, Roche, Manheim, Germany) inhibitors according to manufacturer’s instructions. Total protein (20 μg/sample) was separated with SDS-PAGE using gradient PAGE™ Ex Gels, Mid/High, 4–12% (LONZA, Westburg B.V.) and transferred to PVDF membrane (Immobilon-P transfer membranes, Millipore, Bedford, MA, USA). Membranes were blocked with 5% milk powder in TBS-T (73 mM NaCl, 200 mM Tris HCl, pH = 7.6, and 0.5% Tween 20) for 1 h at room temperature (RT) and blotted overnight at 4°C with primary antibodies, IκBα rabbit monoclonal antibody against NFKBIA (#4,812) (Cell Signaling technology, Danvers, MA, USA) at 1:1,000 final concentration and Actin I-19 goat polyclonal IgG against β-actin (loading control) (sc-1616, Santa-Cruz Biotechnology, Santa Cruz, CA, USA) at final concentration 1:3,000. Blots were incubated with Horseradish Peroxidase (HRP) secondary antibodies (Dako, Glostrup, Danemark), goat anti-rabbit HRP at concentration 1:1,000 against IκBα, and rabbit anti goat-HRP against Actin I-19 at concentration 1:20,000, for 1 h at RT. Specific bands were detected by chemiluminesence using the ECL prime Western Blotting Detection Reagent kit (GE Healthcare Lifesciences, Little Chalfont, UK) and the “ImageQuant LAS4000” (GE Healthcare Lifesciences, Little Chalfont, UK). Intensity of the bands was quantified by optical densitometry using ImageJ software.

### RNA Isolation and Reverse Transcriptase (RT)-PCR

Half of the hypothalamus was homogenized in lysis buffer provided with the “High-Pure RNA isolation Kit” (Roche Molecular Biochemicals, Manheim, Germany) and total RNA was isolated according to the manufacturer’s instructions. RNA was quantified by spectrophotometry at 260 nm (Nanodrop 1000, Wilmington, DE, USA) and 400 ng of total RNA was used per cDNA synthesis reaction using the “Transcriptor First Strand cDNA synthesis kit” for RT-PCR with oligo(dT) primers (Roche Molecular Biochemicals, Manheim, Germany). The mRNA levels of inflammatory and ER stress markers as well as hypoxanthine-guanine phosphoribosyltransferase (*Hprt*) Cyclophilin A (*Ppia*) and β-actin (*Actb*) were determined by RT-PCR using SensiFAST SYBR No-Rox mix (Bioline, Luckenwalde, Germany) at the Lightcycler 480 apparatus (Roche Molecular Biochemicals, Manheim, Germany). Primers against rattus norvegicus specific gene sequences were designed using “Primer Blast” (https://www.ncbi.nlm.nih.gov/tools/primer-blast/) (Table 1) or previously described (20). 2 µl cDNA was added in each PCR reaction. A cDNA synthesis reaction product without RT was used in order to check for genomic DNA contamination and a positive control (purified PCR product) was used to test the accuracy of the PCR. PCR conditions were as follows: denaturation at 95°C for 5 min, amplification (45 cycles) 1 s at 95°C, 10 s at 65°C, and 15 s at 72°C, melting 1 s at 95°C and 15 s at 65°C. 65°C was the melting temperature for most of the primers stated in Table 1 apart from activating transcription factor 4 (*Atf4*) and *usXbp1*, for which it was 70°C. Quantification was performed using the LinRegPCR software, samples were baseline corrected and individually checked for their PCR efficiency using the “LC480 Conversion” and LinRegPCR software. Median efficiency was calculated for each assay and samples that differed more than 0.05 from the mean efficiency were excluded from statistical analysis. Specific gene expression was normalized to the geometric mean of three housekeeping genes (*Hprt* *Actb* *Ppia*)^1/3^ and presented as relative expression.

**Table 1:** Primer sequences used for reverse transcriptase (RT)-PCR.

Primers	Forward 5′–3′	Reverse 5′–3′
*Il1*β	TGTGATGAAAGACGGCACAC	CTTCTTCTTTGGGTATTGTTTGG
*Il6*	TTGTTGACAGCCACTGCCTTCCC	TGACAGTGCATCATCGCTGTTCA
*Tnf*α	AACACACGAGACGCTGAAGT	TCCAGTGAGTTCCGAAAGCC
*Il10*	ATGCAGGACTTTAAGGGTTACTTG	TAGACACCTTGGTCTTGGAGCTTA
*Nfkbia*	AGACTCGTTCCTGCACTTGG	TCTCGGAGCTCAGGATCACA
*Socs3*	CAGCTTTTCGCTGCAGAGTG	CAAAGGAAGGTTCCGTCGGT
*usXbp1*	GTCCGCAGCACTCAGACTAC	ATGAGGTCCCCACTGACAGA
*sXbp1*	CTGAGTCCGAATCAGGTGCAG	ATCCATGGGAAGATGTTCTGG
*Atf4*	CTGAACAGCGAAGTGTTGGC	TCTGTCCCGGAAAAGGCATC
*Ddit3* (CHOP)	AGAGTGGTCAGTGCGCAGC	CTCATTCTCCTGCTCCTTCTCG^a^
*Hspa* (Bip)	TGGGTACATTTGATCTGACTGGA	CTCAAAGGTGACTTCAATCTGGG^a^
*Tlr4*	ATGCCTCTCTTGCATCTGGC	ATTGTCTCAATTTCACACCTGGA
*Hprt*	GCAGTACAGCCCCAAAATGG	AACAAAGTCTGGCCTGTATCCAA
*Ppia*	ATGTGGTCTTTGGGAAGGTG	GAAGGAATGGTTTGATGGGT
*Actb*	CATGTACGTAGCCATCCAGGC	CTCTTTAATGTCACGCACGAT

*^a^Primer pairs for *Ddit3* and *Hsp*α were described by Oslowski and Urano (20)*.

### Statistical Analysis

Differences between LPS and saline or between fcHFHS diet and chow groups were evaluated by two-way ANOVA with two grouping factors (diet and treatment). Only in case of a significant interaction effect of LPS*diet, ANOVA was followed by *post hoc* analysis tests. Mixed–repeated measures ANOVA followed by Bonferroni’s multiple comparisons *post hoc* test was used to compare plasma cytokine and leptin levels at different time points between control and fcHFHS diet groups. Outliers in each group were detected and excluded using Dixon’s Q test. Data were normally distributed in all ANOVA residuals as shown by non-significant Shapiro–Wilk test. Variance was equal between groups according to Levene’s test. *p* < 0.05 was considered significant. All analyses were performed using SPSS (SPSS, Chicago, IL, USA).

## Results

### fcHFHS Diet Does Not Exacerbate LPS-Induced Hypothalamic Cytokine Expression

**Figure 1:**
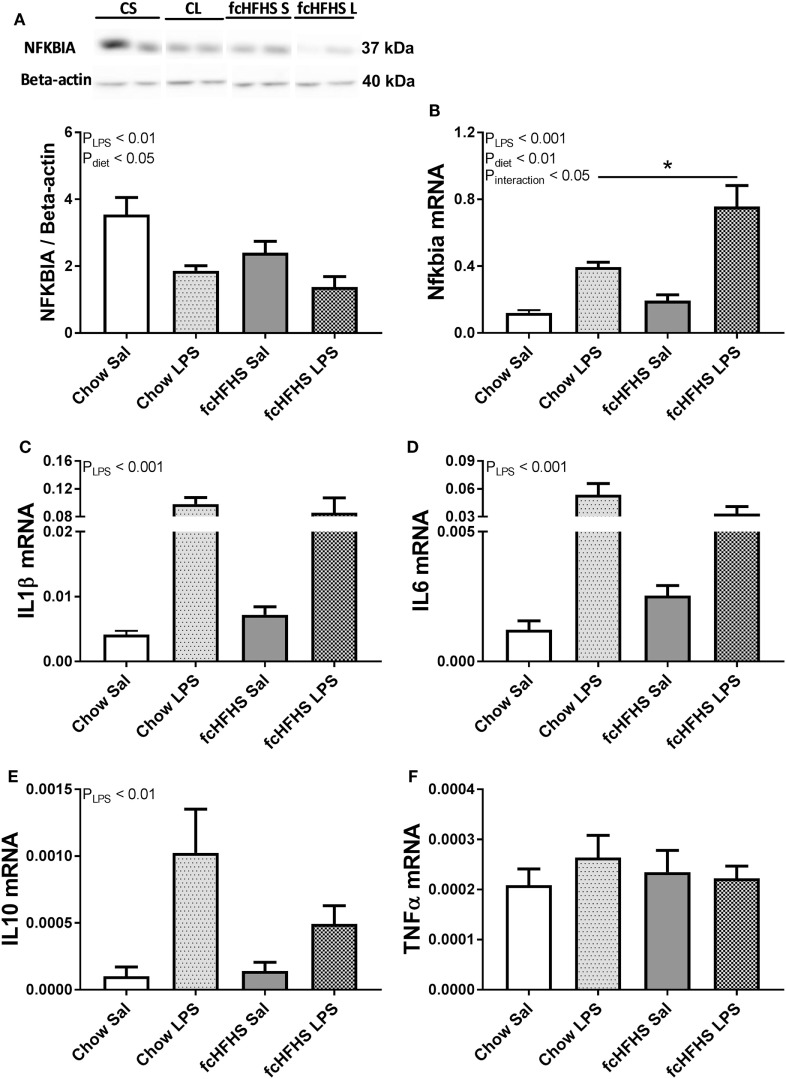
One-week free-choice high-fat high-sugar (fcHFHS) diet and 2 h lipopolysaccharide (LPS) independently reduced hypothalamic **(A)** NFKBIA protein levels and increased **(B)** relative *Nfkbia* mRNA expression in rats. Blots of NFKBIA (Figure S1 in Supplementary Material) and Beta-actin (Figure S2 in Supplementary Material) in **(A)** include two bands per group (CS, chow saline; CL, chow LPS; fcHFHS S, fcHFHS saline; fcHFHS L, fcHFHS LPS) that represents significant effects seen in eight animals per group. **(C)** Relative *Il1*β, **(D)** *Il6*, and **(E)** *Il10* mRNA expression was increased only upon LPS, whereas **(F)** relative *Tnf*α mRNA expression did not change. Statistical analysis was performed using two-way ANOVA followed by *post hoc* analysis in case of a significant interaction effect. Specific gene expression was normalized to the geometric mean of three housekeeping genes; (*Hprt* *Actb* *Ppia*)^1/3^. Data are presented as mean (*n* = 8) ± SEM; *p < 0.05.

Animals on 1 -week fcHFHS diet displayed increased average daily caloric intake and % white adipose tissue relative to body weight (%WAT/BW) compared with chow while ΔBW was not different between the groups (Table 2). LPS administration and fcHFHS diet independently resulted in decreased NFKBIA protein levels (Figures 1A,B), whereas the *Nfkbia* mRNA response to LPS was more severe in animals on the fcHFHS diet compared with chow-fed animals. LPS administration significantly increased hypothalamic *Il1*β, *Il6*, and *Il10* mRNA expression irrespective of whether animals were consuming chow or the fcHFHS diet (Figures 1C–E), and hypothalamic *Tnf*α mRNA expression did not change in any group (Figure 1F). mRNA expression of the hypothalamic microglia marker *iba1* did not change upon LPS and diet while *CD11b* increased after 1 week fcHFHS diet (ANOVA *p*_diet_ < 0.05, data not shown).

**Table 2:** Metabolic characteristics of animals in experiments 1 and 2.

Animals ex1	Chow sal	Chow LPS	fcHFHS sal	fcHFHS LPS
ΔBW (g)	34 ± 1.2	35 ± 0.8	37 ± 1.1	39 ± 1.0
% WAT/BW	2.58 ± 0.05	2.63 ± 0.04	3.41 ± 0.03****	3.31 ± 0.05****
Average consumption/day (kcal)	76 ± 1.5	74 ± 1.3	100 ± 2.0****	101 ± 1.5****

**Animals ex2**	**Chow**		**fcHFHS**	

ΔBW (g)	30 ± 0.9		35 ± 0.8*	
% WAT/BW	2.42 ± 0.04		3.32 ± 0.05****	
Average consumption/day (kcal)	53 ± 1.0		79 ± 2.6****	

Delta body weight, % total white adipose tissue (WAT) normalized to final body weight and average daily caloric intake in chow-fed and free-choice high-fat high-sugar (fcHFHS)-fed cohorts from experiment 1 (*n* = 8/group) and experiment 2 (*n* = 10/group). Total WAT represents the sum of mesenteric, peritoneal, subcutaneous, and epididymal fat; two-way ANOVA followed by *post hoc* analysis was used to determine significant differences between groups, *p < 0.05, ****p < 0.0001.

### fcHFHS Diet and LPS Induce Different Signaling Pathways

**Figure 2:**
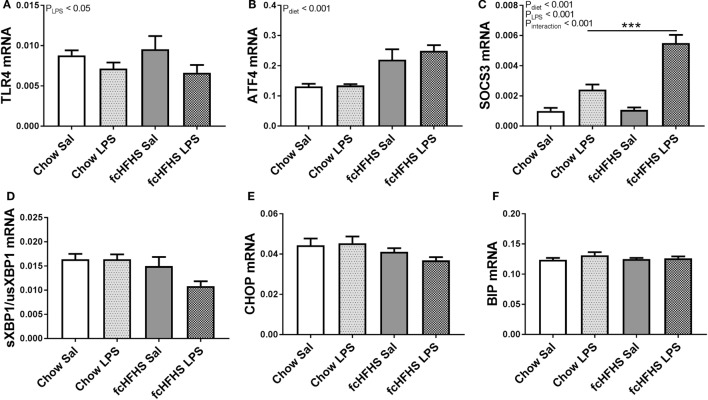
Lipopolysaccharide (LPS) decreased **(A)** relative toll-like receptor 4 (*Tlr4*) mRNA expression, whereas 1 week of free-choice high-fat high-sugar (fcHFHS) diet increased **(B)** relative activating transcription factor 4 (*Atf4*) mRNA expression in the hypothalamus of rats. An interaction effect between diet and LPS was observed in **(C)** relative suppressor of cytokine signaling (Socs) 3 mRNA levels. Relative expression of endoplasmic reticulum stress markers **(D)** *sXBP1*/*usXBP1*, **(E)** C/EBP homologous protein (CHOP) or **(F)** immunoglobulin-heavy-chain-binding protein (BIP) were not affected. Statistical analysis was performed using two-way ANOVA followed by *post hoc* analysis in case of an interaction effect. Specific gene expression was normalized to the geometric mean of three housekeeping genes (*Hprt* *Actb* *Ppia*)^1/3^. Data are presented as mean (*n* = 8) ± SEM; ***p < 0.001.

Toll-like receptor 4 (*Tlr4*) mRNA expression was only affected by LPS and not by diet (Figure 2A). Furthermore, it has been shown that ER stress can be activated during a high-fat diet, which also leads to NF-κB activation (23, 24). The cellular stress marker *Atf4* was increased in the hypothalamus of rats after 1-week fcHFHS diet exposure and not affected by LPS administration (Figure 2B). Other markers of cellular stress, such as the ratio of the spliced transcript variant of X box binding protein 1 (*sXbp1*) to the unspliced variant (*usXbp1*), C/EBP homologous protein (CHOP), and immunoglobulin-heavy-chain-binding protein (BiP) mRNA expression were not affected by LPS or diet (Figures 2C–E). Ultimately, hypothalamic *Socs3* mRNA levels increased upon LPS injections in both the fcHFHS diet and chow group, however, the response to LPS was more pronounced in fcHFHS diet-fed rats (Figure 2F).

### LPS Administration to Rats on an fcHFHS Diet Does Not Exacerbate the Acute Phase Response

**Figure 3:**
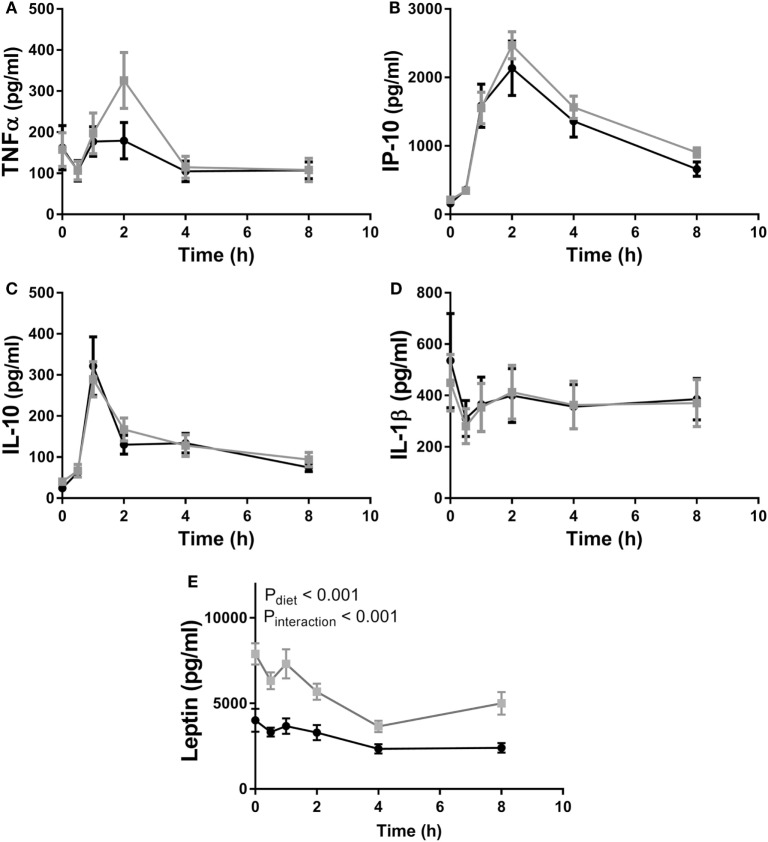
Plasma **(A)** TNFα, **(B)** IP10, and **(C)** IL10 increased after lipopolysaccharide (LPS) administration in both chow (black circles) and free-choice high-fat high-sugar diet (light squares) animals, whereas **(D)** IL1β levels remained stable during the time course of the experiment. Diet had no effect on plasma cytokine levels. An interaction effect between diet and LPS was observed in **(E)** serum leptin levels. Mixed–repeated measures ANOVA followed by Bonferroni’s multiple comparisons *post hoc* analysis was used to determine significant differences in cytokine levels between different time points and diet groups. Data are presented as mean (*n* = 10) ± SEM.

In experiment 2, as in experiment 1, fcHFHS diet-fed animals displayed higher average daily consumption followed by increased %WAT/BW compared with chow controls (Table 2). In addition, we also observed an increase in ΔBW of fcHFHS diet-fed rats compared with chow-fed rats. LPS administration resulted in a rapid increase of inflammatory cytokines in the circulation with a peak at 1 h for IL10 and 2 h for IP10 in both groups. Eight hours after LPS all cytokines returned to baseline levels. No changes in IL1β, TNFα, IL10, and IP10 plasma levels were observed between the fcHFHS diet-fed and chow-fed animals after LPS administration (Figure 3). Leptin levels were higher in fcHFHS diet-fed rats compared with chow-fed rats and responded differently to LPS (Figure 3).

## Discussion

The aim of the present study was to determine whether the higher susceptibility to infection, shown to occur in obesity is mediated by diet-induced hypothalamic low-grade inflammation. We recently showed that animals exposed to a fcHFHS diet for only 1-week develop low-grade hypothalamic inflammation characterized by decreased hypothalamic NFKBIA protein and increased *Nfkbia* and *Il6* mRNA, before body weight gain is apparent (20). This model provides us with the possibility to test whether mild hypothalamic inflammation, which is already present at the time of LPS administration, aggravates the LPS-induced inflammatory response. The present study showed that both LPS administration and the fcHFHS diet decreased hypothalamic NFKBIA protein and increased *Nfkbia* mRNA. The LPS-induced increase in *Nfkbia* mRNA was affected by the diet as *Nfkbia* mRNA was significantly higher in animals on a fcHFHS diet compared with chow controls. The previously observed increase in hypothalamic *Il6* mRNA expression did not reach statistical significance due to the experimental set up which was focused on interaction effects between diet and LPS and did not include *post hoc* analysis between chow/control and diet/control groups.

Despite the difference in hypothalamic *Nfkbia* mRNA, LPS administration to rats exposed to the fcHFHS diet did not induce a more pronounced hypothalamic cytokine response compared with rats on chow. This is in contrast to the study of Pohl et al., as they observed higher hypothalamic *Il1*β, *Il6*, and *Tnf*α mRNA expression upon the same amount of LPS administered to rats on a high-fat diet compared with those on chow diet (6). These alterations were associated with a prolonged fever and increased expression of cytokines in adipose tissue. However, those rats were exposed to a palatable diet for a prolonged period and gained significant body weight which was not observed in our rats on the fcHFHS diet for 1 week (in experiment 1). In general, these data suggest that the hypothalamic inflammatory mediators activated by consuming the fcHFHS diet did not interact with the inflammatory pathways induced by the acute phase response but points to involvement of additional factors other than nutrients, occurring within the obesity state, in the acute phase response susceptibility.

Lipopolysaccharide activates brain microglia *via* the TLR4/NF-κB pathway (25). In turn, TLR4 activation increases *Socs3* mRNA expression (26). Interestingly, we observed an increase in hypothalamic *Socs3* mRNA expression after LPS administration which was exacerbated in rats on the fcHFHS diet. This interaction effect of diet and LPS on *Socs3* mRNA expression could be due to higher plasma leptin concentrations, observed in rats on the fcHFHS diet. Leptin is known to increase *Socs3* mRNA expression *via* the janus kinase/signal transducer and activator of transcription pathway (27). However, increased leptin levels observed in fcHFHS diet-fed animals were not sufficient to alter the systemic acute phase response or induce an interaction effect of diet and LPS in hypothalamic NFKBIA protein expression. Given the fact that LPS induces inflammatory signaling exclusively *via* the TLR4 receptor (28, 29) and both diet and LPS independently affected the NF-κB response, we further aimed to identify additional signal transduction pathways involved in the diet-induced hypothalamic inflammation signaling. We recently showed that 1-week exposure to the fcHFHS diet resulted in increased mRNA expression of cellular stress markers (30). We therefore measured *Atf4*, unspliced and spliced variant of *Xbp1, CHOP*, and *BiP* mRNA that represent the activation of the unfolded protein response (31). We showed that *Atf4* mRNA was only increased by the fcHFHS diet. However, *sXbp1* and *BiP* mRNA, well-known ER stress markers (32, 33), as well as *CHOP* that was shown to be induced downstream of ATF4 (34, 35) were not affected after 1 week of fcHFHS diet suggesting that ATF4 can be seen as a general cell stress marker due to fat consumption (30). ATF4 is known to be able to activate NF-κB (36) and might therefore be responsible for the activation of hypothalamic NF-κB after 1-week fcHFHS diet exposure. This is also supported by the fact that cellular stress was shown to be the exclusive mechanism induced in cultured hypothalamic neurons in response to palmitate (37).

Finally, we showed that LPS administration results in an acute phase response characterized by a rapid increase in circulating TNFα, IL10, IP10 in both fcHFHS diet-fed and chow-fed animals. The magnitude of the cytokine response was similar in fcHFHS diet-fed animals compared with chow-fed animals.

Although the animals were only exposed to the fcHFHS diet for a short period, it did lead to small but significant increases in adiposity associated with elevated plasma leptin concentrations. We cannot conclude therefore that the effects of the fcHFHS diet on hypothalamic inflammation are completely independent of metabolic changes, although the changes shown after 1 week diet were not enough to alter the acute phase response. To further unravel the role of nutrients and metabolic parameters on hypothalamic inflammation, direct effects of nutrients on the brain could be studied without affecting the peripheral metabolic parameters by, for example, using the carotid brain catheter technique we recently described which enables infusion of nutrients directly toward the brain (38). To further unravel the role of obesity and its metabolic effects on hypothalamic inflammation and the acute phase response, animals could be exposed to different obesogenic diets (using fat and/or sugar to enrich the diets) for a prolonged period of time and then changes in adipose tissue, macrophages, liver, and spleen could be determined, and the role of the observed changes in the acute phase response. One might also use genetically induced obese models and compare this with DIO.

In conclusion, LPS administration to rats on a fcHFHS diet for 1 week did not result in an exaggerated systemic acute phase response or a larger hypothalamic inflammatory response. Moreover, changes in the mRNA of the cellular stress marker *Atf4* showed that alterations in hypothalamic NFKBIA protein and *Nfkbia* mRNA levels observed when consuming a fcHFHS diet or after LPS administration might be due to the induction of different pathways. Our results do not support the hypothesis that diet-induced hypothalamic NF-κB activation contributes to an exacerbated acute phase response induced by LPS.

## Ethics Statement

All the studies were approved by and performed according to the regulations of the Committee for Animal Experimentation of the Academic Medical Centre of the University of Amsterdam, Netherlands.

## Author Contributions

EB, SF, and AB designed experiments and prepared the manuscript. EB and LE performed experiments. LE edited the manuscript. The entire study was supervised by SF and AB.

## Conflict of Interest Statement

The authors declare that the research was conducted in the absence of any commercial or financial relationships that could be construed as a potential conflict of interest.

## Supplementary Material

Figure S1 Protein expression of nuclear factor of kappa light polypeptide gene enhancer in B cells inhibitor alpha (NFκBIA) in hypothalamic homogenates of rats after one week on free-choice high-fat high-sugar (fcHFHS) or chow diet and 2 h lipopolysaccharide (LPS) or saline (IP) administration. Blots were incubated with IκBα rabbit monoclonal antibody against NFκBIA and goat anti rabbit-horseradish peroxidase against IκBα. Specific bands were detected by chemiluminescence, and the intensity of bands was quantified by optical densitometry.

Figure S2 Protein expression of beta actin in hypothalamic homogenates of rats after 1 week on free-choice high-fat high-sugar (fcHFHS) or chow diet and 2 h lipopolysaccharide (LPS) or saline (IP) administration. Blots were incubated with Actin I-19 goat polyclonal IgG against beta actin and rabbit anti goat-horseradish peroxidase against Actin I-19. Specific bands were detected by chemiluminescence, and the intensity of bands was quantified by optical densitometry.

## Abbreviations

ATF4, activating transcription factor 4; BiP, immunoglobulin-heavy-chain-binding protein; CHOP, C/EBP homologous protein; ER, endoplasmic reticulum; fcHFHS, free-choice high-fat high-sugar; IP, intraperitoneal; LPS, lipopolysaccharide; NF-κB, nuclear factor kappa-light-chain-enhancer of activated B cells.
