# Supplementary figures and images for: One-Week Exposure to a Free-Choice High-Fat High-Sugar Diet Does Not Interfere With the Lipopolysaccharide-Induced Acute Phase Response in the Hypothalamus of Male Rats
Source: Front Endocrinol (Lausanne). 2018 Apr 30;9:186. doi: 10.3389/fendo.2018.00186 (PMC5937016; doi:10.3389/fendo.2018.00186)

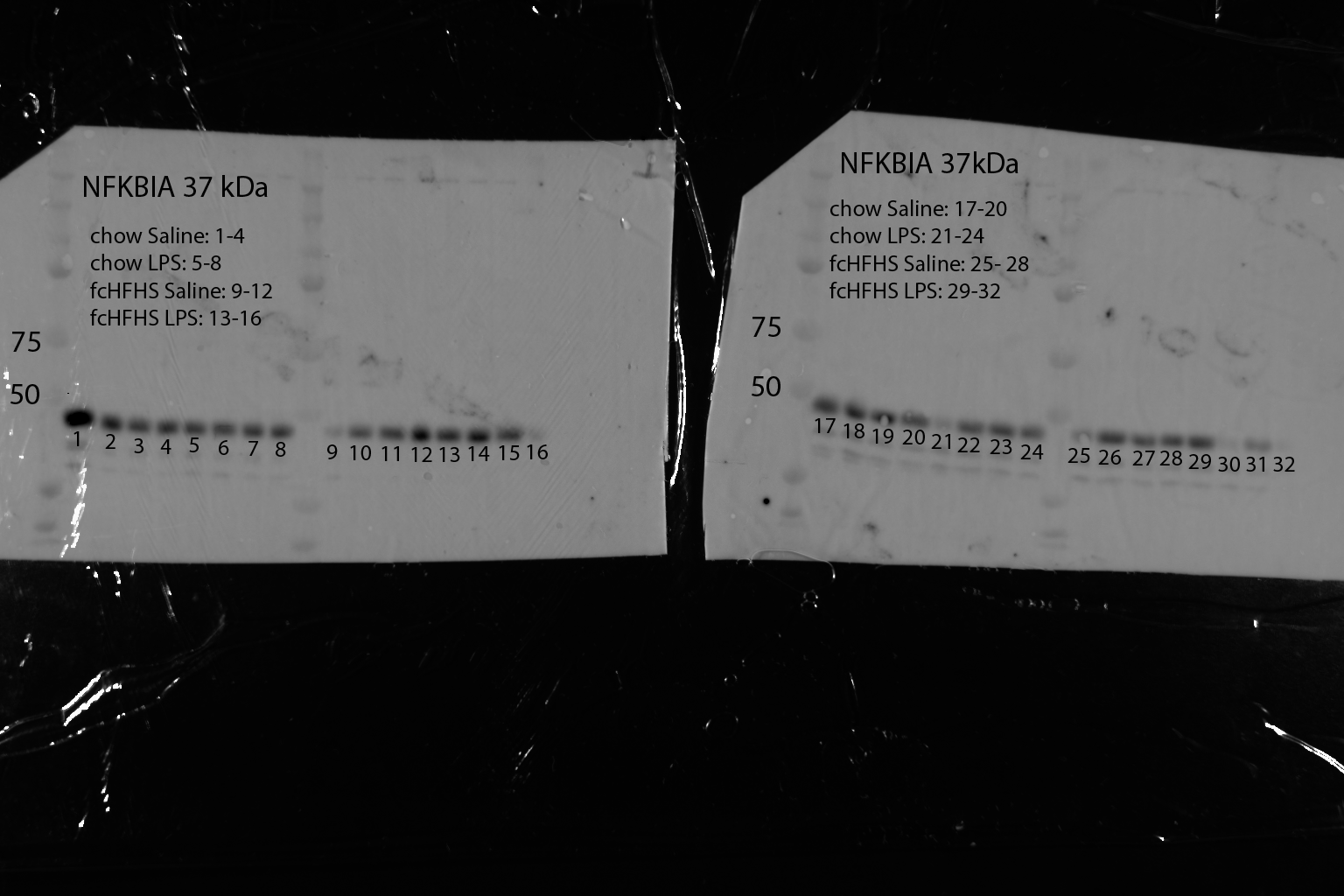

Supplement: Figure S1 — Protein expression of nuclear factor of kappa light polypeptide gene enhancer in B cells inhibitor alpha (NFκBIA) in hypothalamic homogenates of rats after one week on free-choice high-fat high-sugar (fcHFHS) or chow diet and 2 h lipopolysaccharide (LPS) or saline (IP) administration. Blots were incubated with IκBα rabbit monoclonal antibody against NFκBIA and goat anti rabbit-horseradish peroxidase against IκBα. Specific bands were detected by chemiluminescence, and the intensity of bands was quantified by optical densitometry. [file Image_1.TIF]
